# Supplementary material for: Live-cell tracking of biliverdin trafficking reveals metabolic exchange between plastids and peroxisomes
Source: Biol Open. 2026 May 18;15(5):bio062435. doi: 10.1242/bio.062435 (PMC13225713; doi:10.1242/bio.062435)
Supplement: Supplementary information [file biolopen-15-062435-s1.pdf]

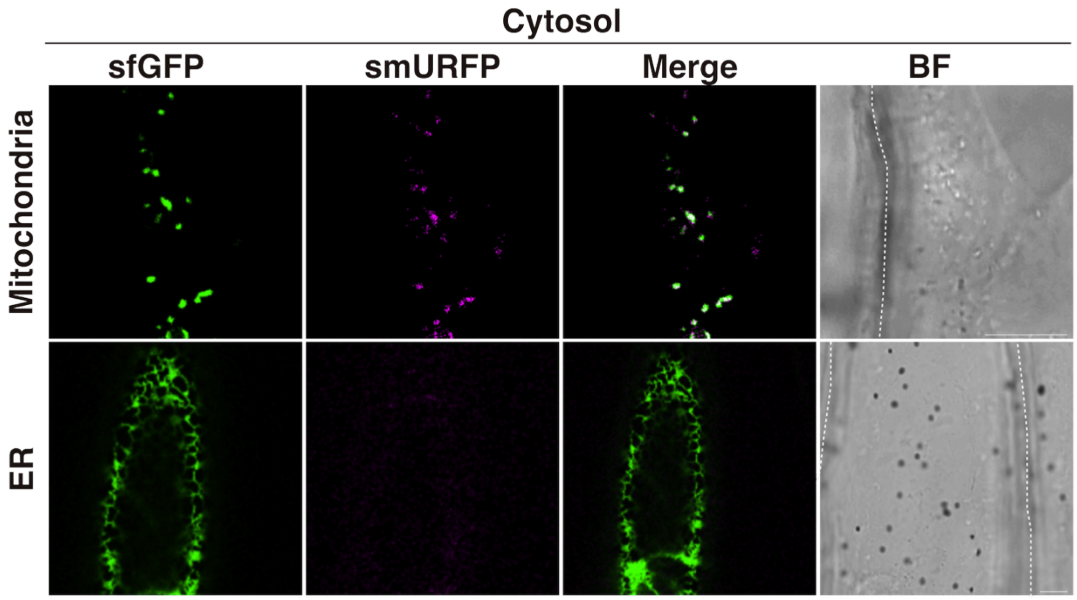

Fig. S1. Endogenous BV was detected by smURFP in mitochondria but not in the ER. Representative images of sfGFP and smURFP in mitochondria and ER of onion cells expressing sfGFP-smURFP probes targeted to the mitochondria ($MT^{sfGFP\text{-}smURFP}$) or ER ($ER^{sfGFP\text{-}smURFP}$), respectively. Bars, 20 µm. BF, bright field. Cell shapes are traced with dashed lines in BF images.

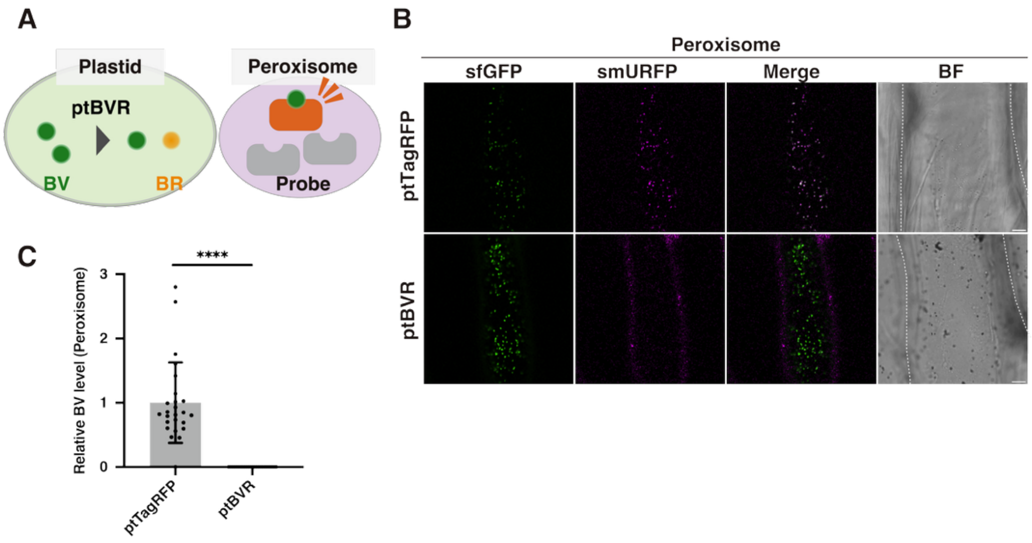

Fig. S2. Lower BV levels in peroxisomes of cells expressing ptBVR. (A) Illustration of *trans*-organelle assessment with $PS^{sfGFP\text{-}smURFP}$ in peroxisomes and ptBVR in plastids. BR, bilirubin; BV, biliverdin; BVR, BILIVERDIN REDUCTASE; probe, sfGFP-smURFP. (B) Representative images of sfGFP and smURFP in peroxisomes of onion cells expressing $PS^{sfGFP\text{-}smURFP}$ probe and ptBVR. Bars, 20 µm. BF, bright field. Cell shapes are traced with dashed lines in BF images. (C) Lower relative BV levels in peroxisomes of cells expressing ptBVR. BV levels were quantified using the fluorescence intensities of smURFP and sfGFP (see Methods). The mean BV level in ptTagRFP-expressing cells was set to 1, and the relative BV level in ptBVR-expressing cells was determined. Fluorescence intensities from 25 peroxisomes of five onion cells (five peroxisomes each) were measured. Values are means ± standard deviations (n = 25). Asterisks indicate significant differences (Student’s t-test, ****$p < 0.0001$).

Biology Open • Supplementary information

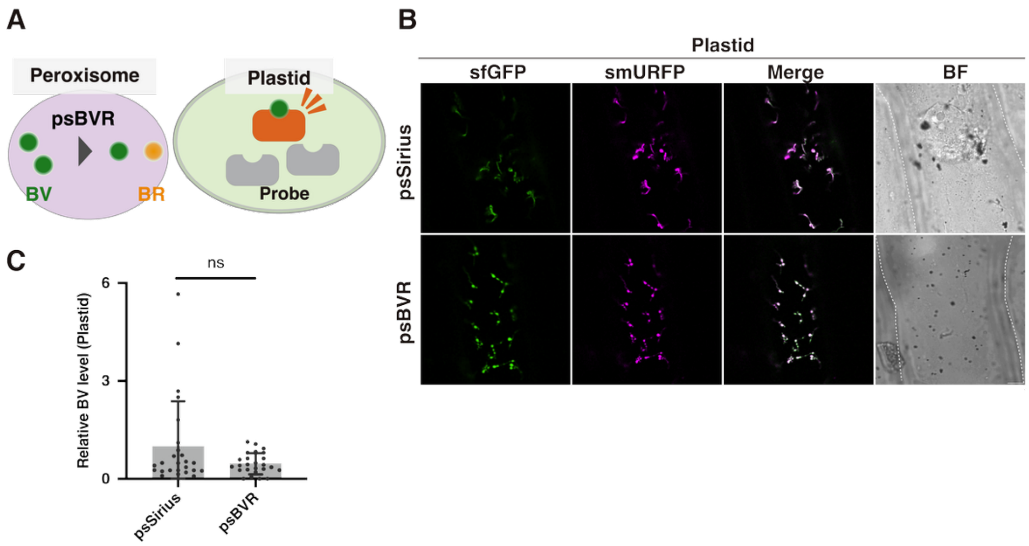

Fig. S3. Comparable BV levels in plastids of cells expressing psBVR. (A) Illustration of *trans*-organelle assessment with PT$^{sfGFP\text{-}smURFP}$ in plastids and psBVR in peroxisomes. BR, bilirubin; BV, biliverdin; BVR, BILIVERDIN REDUCTASE; probe, sfGFP-smURFP. (B) Representative images of sfGFP and smURFP in plastids of onion epidermal cells expressing PT$^{sfGFP\text{-}smURFP}$ probe and psBVR. Bars, 20 µm. BF, bright field. Cell shapes are traced with dashed lines in BF images. (C) Comparable relative BV levels in plastids of cells expressing psBVR. BV levels were quantified using the fluorescence intensities of smURFP and sfGFP (see Methods). The mean BV level in psSirius-expressing cells was set to 1, and the relative BV level in psBVR-expressing cells was determined. Fluorescence intensities from 25 plastids of five onion cells (five plastids each) were measured. Values are means ± standard deviations (n = 25). ns, No significant difference (Student's t-test, $p > 0.05$).

Biology Open • Supplementary information

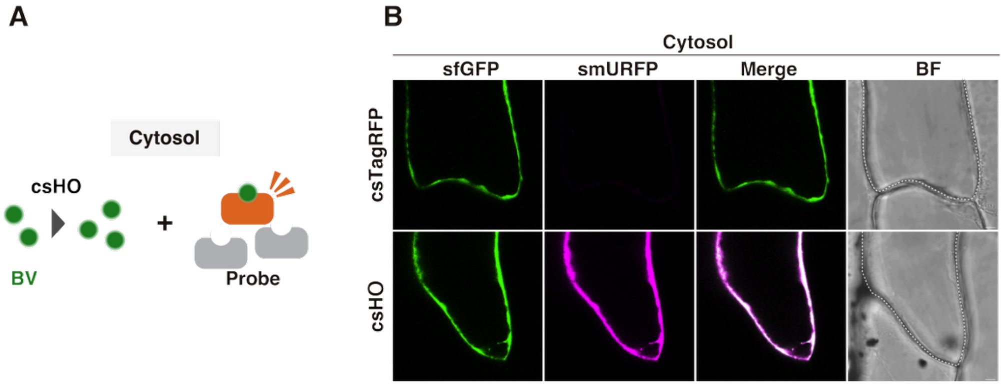

Fig. S4. Higher BV levels in the cytosol of cells expressing csHO. (A) Illustration of an experiment with CS$^{sfGFP\text{-}smURFP}$ and csHO in the cytosol. BV, biliverdin; HO, HEME OXYGENASE; probe, sfGFP-smURFP. (B) Representative images of sfGFP and smURFP in the cytosol of onion cells expressing CS$^{sfGFP\text{-}smURFP}$ probe and csHO. Bars, 20 µm. BF, bright field. Cell shapes are traced with dashed lines in BF images.

Biology Open • Supplementary information

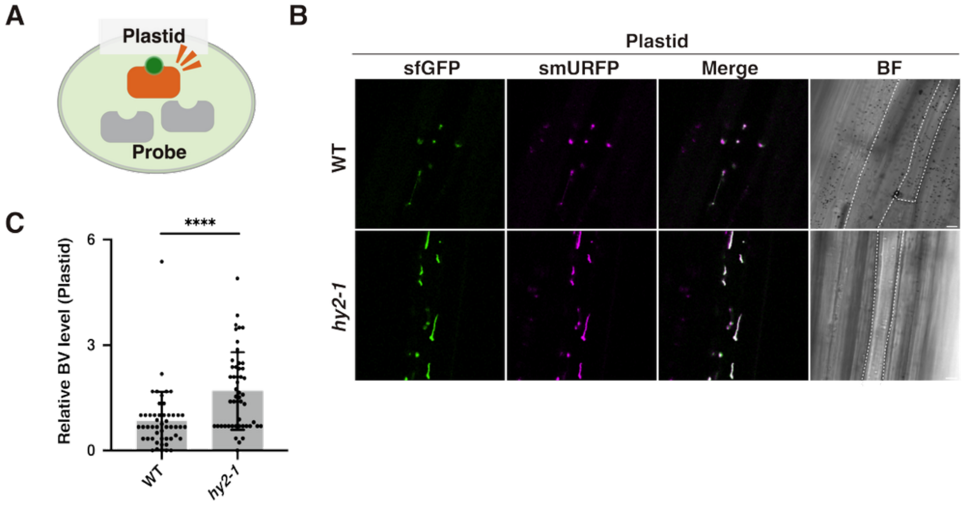

Fig. S5. smURFP fluorescence in Arabidopsis wild-type and *hy2-1* mutants. (A) Illustration of an experiment with PT$^{sfGFP\text{-}smURFP}$ in the plastid. Probe, sfGFP-smURFP. (B) Representative images of sfGFP and smURFP in the plastid of wild-type (WT; Col-0) and *hy2-1* mutant cells expressing PT$^{sfGFP\text{-}smURFP}$ probe. The *hy2-1* mutant line (Kohchi *et al*, 2001) was obtained from the Arabidopsis Biological Resource Center (CS2068). Arabidopsis seeds were surface sterilized and sown on half-strength Murashige and Skoog medium supplemented with 0.5% (w/v) gellan gum and 1% (w/v) sucrose. For germination, the seeds were incubated in the dark for 3 days at 4°C and then exposed to continuous white light (50 µmol m^{-2} s^{-1}) for 24 h. Seedlings were then grown vertically in the dark for 3–5 days at 22°C. The resulting dark-grown seedlings were subjected to particle bombardment. Bars, 20 µm. BF, bright field. Cell shapes are traced with dashed lines in BF images. (C) Relative BV levels in plastids of WT and *hy2-1* cells. BV levels were quantified using the fluorescence intensities of smURFP and sfGFP (see Methods). The mean BV level in WT cells was set to 1, and the relative BV level in *hy2- 1* cells was determined. Fluorescence intensities from 50 plastids of five seedlings (10 plastids each) were measured. Values are means ± standard deviations (n = 50). Asterisks indicate significant differences (Student's t-test, ****$p < 0.0001$).

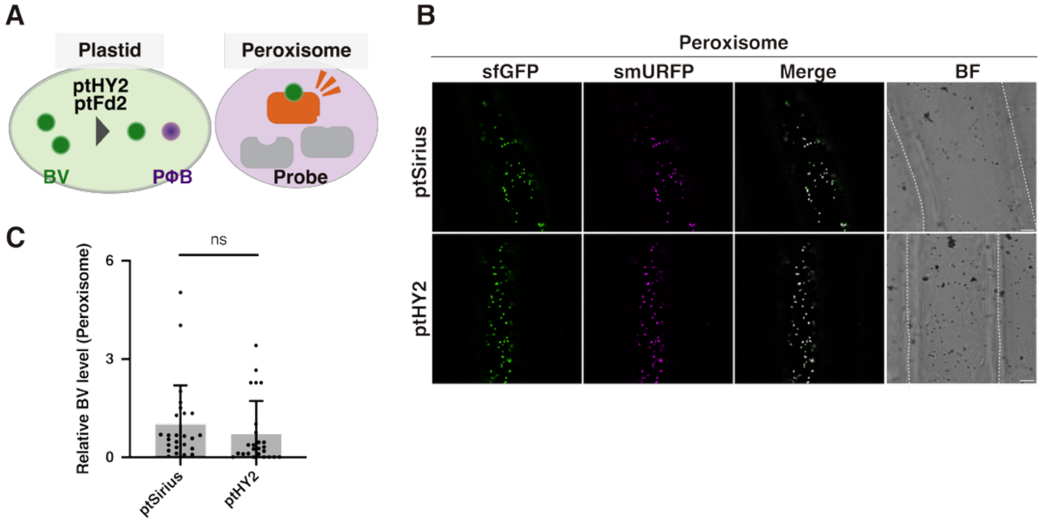

Fig. S6. Comparable BV levels in peroxisomes of cells expressing ptHY2. (A) Illustration of *trans*-organelle assessment with $PS^{sfGFP\text{-}smURFP}$ in peroxisomes and ptHY2 in plastids. BV, biliverdin; probe, sfGFP-smURFP; ptFd2, FERREDOXIN 2; ptHY2, PΦB SYNTHASE; PΦB, phytochromobilin. (B) Representative images of sfGFP and smURFP in peroxisomes of onion cells expressing $PS^{sfGFP\text{-}smURFP}$ probe and ptHY2. Bars, 20 µm. BF, bright field. Cell shapes are traced with dashed lines in BF images. (C) Relative BV levels in peroxisomes of cells expressing ptHY2 with ptFd2. BV levels were quantified using the fluorescence intensities of smURFP and sfGFP (see Methods). The mean BV level in ptSirius-expressing cells was set to 1, and the relative BV level in ptHY2-expressing cells was determined. Fluorescence intensities from 25 peroxisomes of five onion cells (five peroxisomes each) were measured. Values are means ± standard deviations (n = 25). ns, no significant difference (Student's t-test, $p > 0.05$).

Biology Open • Supplementary information

```
   1 ATGGCTTCCT CTATGCTCTC TTCCGCTACT ATGGTTGCCT CTCCGGCTCA GGCCACTATG
  61 GTCGCTCCTT TCAACGGACT TAAGTCCTCC GCTGCCTTCC CAGCCACCCG CAAGGCTAAC
 121 AACGACATTA CTTCCATCAC AAGCAACGGC GGAAGAGTTA ACTGCATGCA GGTGTGGCCT
 181 CCGATTGGAA AGAAGAAGTT TGAGACTCTC TCTTACCTTC CTGACCTTAC CGATTCCATG
 241 GTGAGCAAGG GCGAGGAGCT GTTCACCGGG GTGGTGCCCA TCCTGGTCGA GCTGGACGGC
 301 GACGTAAACG GCCACAAGTT CAGCGTGAGA GGCGAGGGCG AGGGCGATGC CACCAACGGC
 361 AAGCTGACCC TGAAGTTCAT CTGCACCACC GGCAAGCTGC CCGTGCCCTG GCCCACCCTC
 421 GTGACCACCC TGACCTACGG CGTGCAGTGC TTCAGCCGCT ACCCCGACCA CATGAAGCAG
 481 CACGACTTCT TCAAGTCCGC CATGCCCGAA GGCTACGTCC AGGAGCGCAC CATCTCTTTC
 541 AAGGACGACG GCACTTACAA GACCCGCGCC GAGGTGAAGT TCGAGGGCGA CACCCTGGTG
 601 AACCGCATCG AGCTGAAGGG CATCGACTTC AAGGAGGACG GCAACATCCT GGGGCACAAG
 661 CTGGAGTACA ACTTCAACAG CCACAACGTC TATATCACTG CCGACAAGCA GAAGAACGGC
 721 ATCAAGGCCA ACTTCAAGAT CCGCCACAAC GTTGAGGACG GCAGCGTGCA GCTCGCCGAC
 781 CACTACCAGC AGAACACCCC CATCGGCGAC GGCCCCGTGC TGCTGCCCGA CAACCACTAC
 841 CTGAGCACCC AGTCCAAGCT GAGCAAAGAC CCCAACGAGA AGCGCGATCA CATGGTCCTG
 901 CTGGAGTTCG TGACCGCCGC CGGGATCACT CTCGGCATGG ACGAGCTGTA CAAGGGCGGT
 961 AGCGGCGGTA TGGCTAAGAC TTCCGAACAG AGGGTGAACA TTGCTACACT GCTGACAGAA
1021 AATAAGAAGA AAATCGTGGA TAAGGCTTCC CAGGATCTGT GGCGGAGACA CCCAGACCTG
1081 ATCGCACCAG GAGGAATTGC TTTCTCTCAG AGGGACCGCG CTCTGTGCCT GCGAGATTAC
1141 GGCTGGTTCC TGCATCTGAT CACCTTTTGT CTGCTGGCCG GAGATAAGGG CCCCATCGAG
1201 TCTATTGGGC TGATCAGTAT TCGAGAAATG TATAACTCAC TGGGAGTGCC CGTCCCTGCA
1261 ATGATGGAGA GCATTAGATG CCTGAAAGAA GCCAGCCTGT CCCTGCTGGA CGAAGAGGAC
1321 GCCAACGAGA CCGCACCCTA CTTTGATTAC ATTATTAAGG CTATGAGCTA A
```

Fig. S7. DNA sequence encoding PT$^{sfGFP\text{-}smURFP}$. Gray, green, and pink highlight the regions encoding a plastid-targeting peptide (N-terminal 79 amino acids of ribulose bisphosphate carboxylase small chain 1A from Arabidopsis), sfGFP, and smURFP, respectively.

Biology Open • Supplementary information

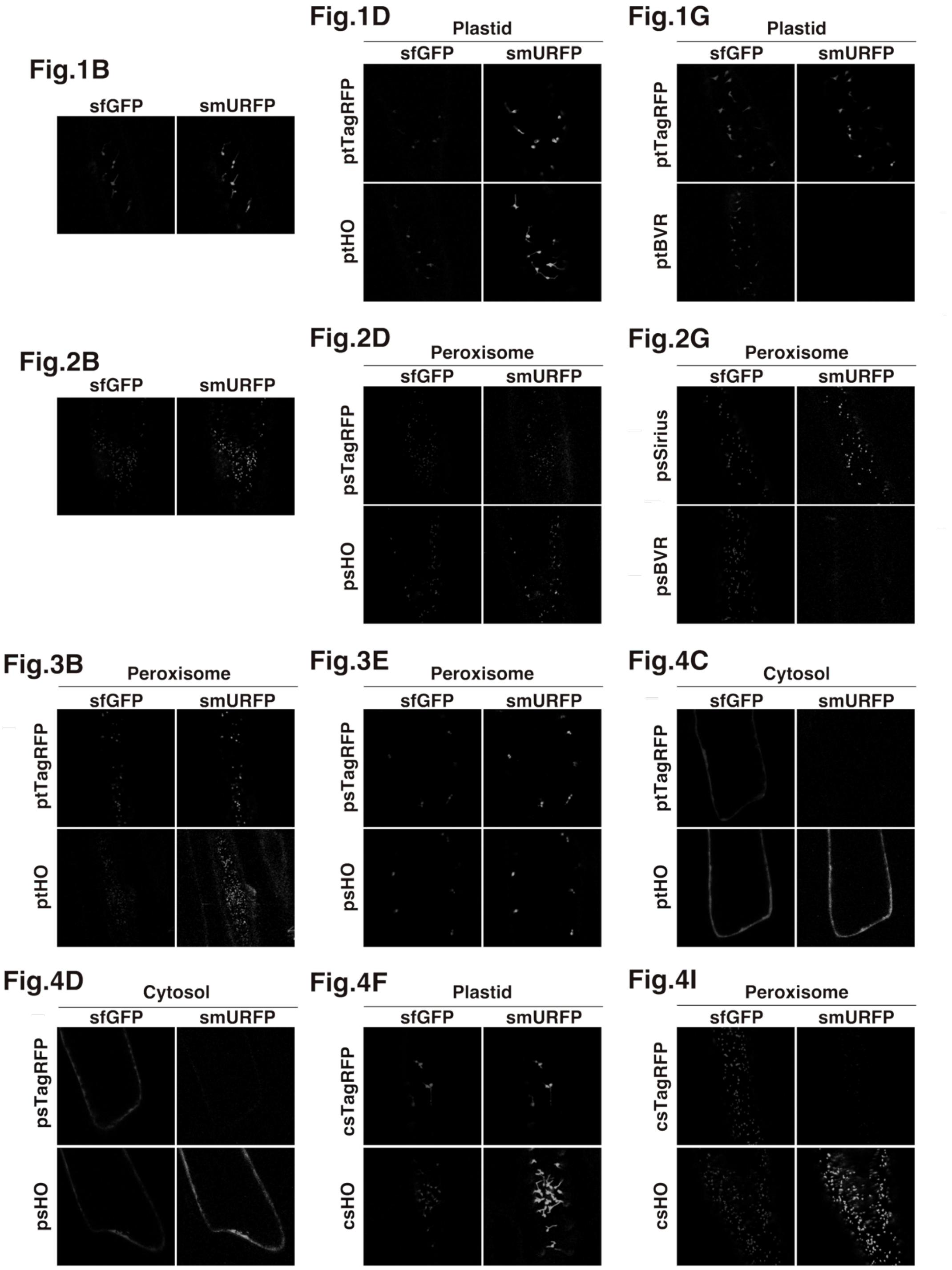

Fig. S8. Grayscale images of sfGFP and smURFP in Figs. 1–4. Grayscale images corresponding to Figs. 1B, 1D, 1G, 2B, 2D, 2G, 3B, 3E, 4C, 4D, 4F, and 4I are shown.

Biology Open • Supplementary information

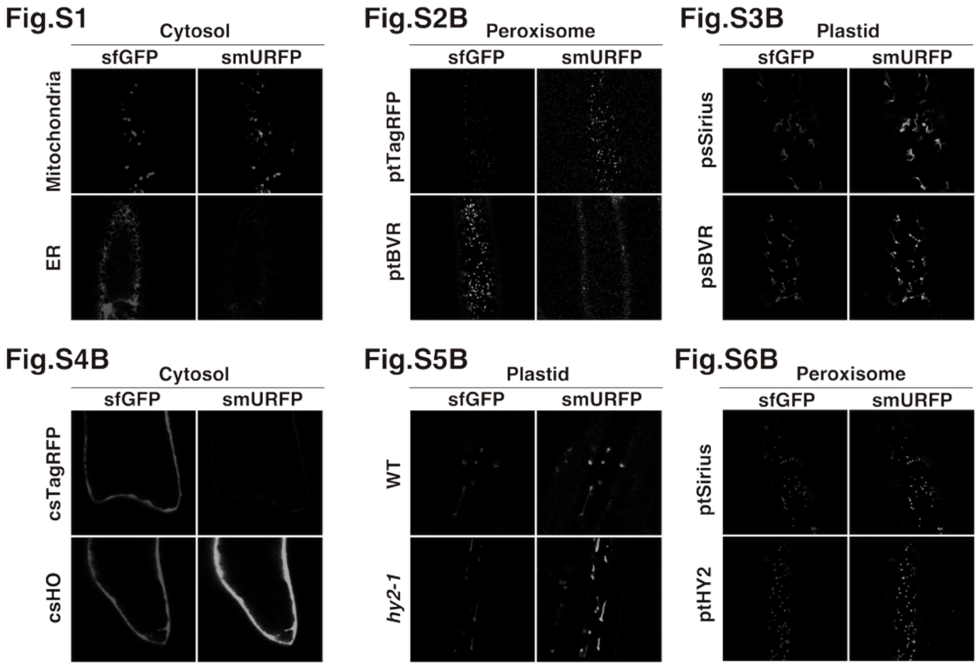

Fig. S9. Grayscale images of sfGFP and smURFP in Supplementary Figs. S1–S6. Grayscale images corresponding to Supplementary Figs. S1, S2B, S3B, S4B, S5B, and S6B are shown.

Biology Open • Supplementary information

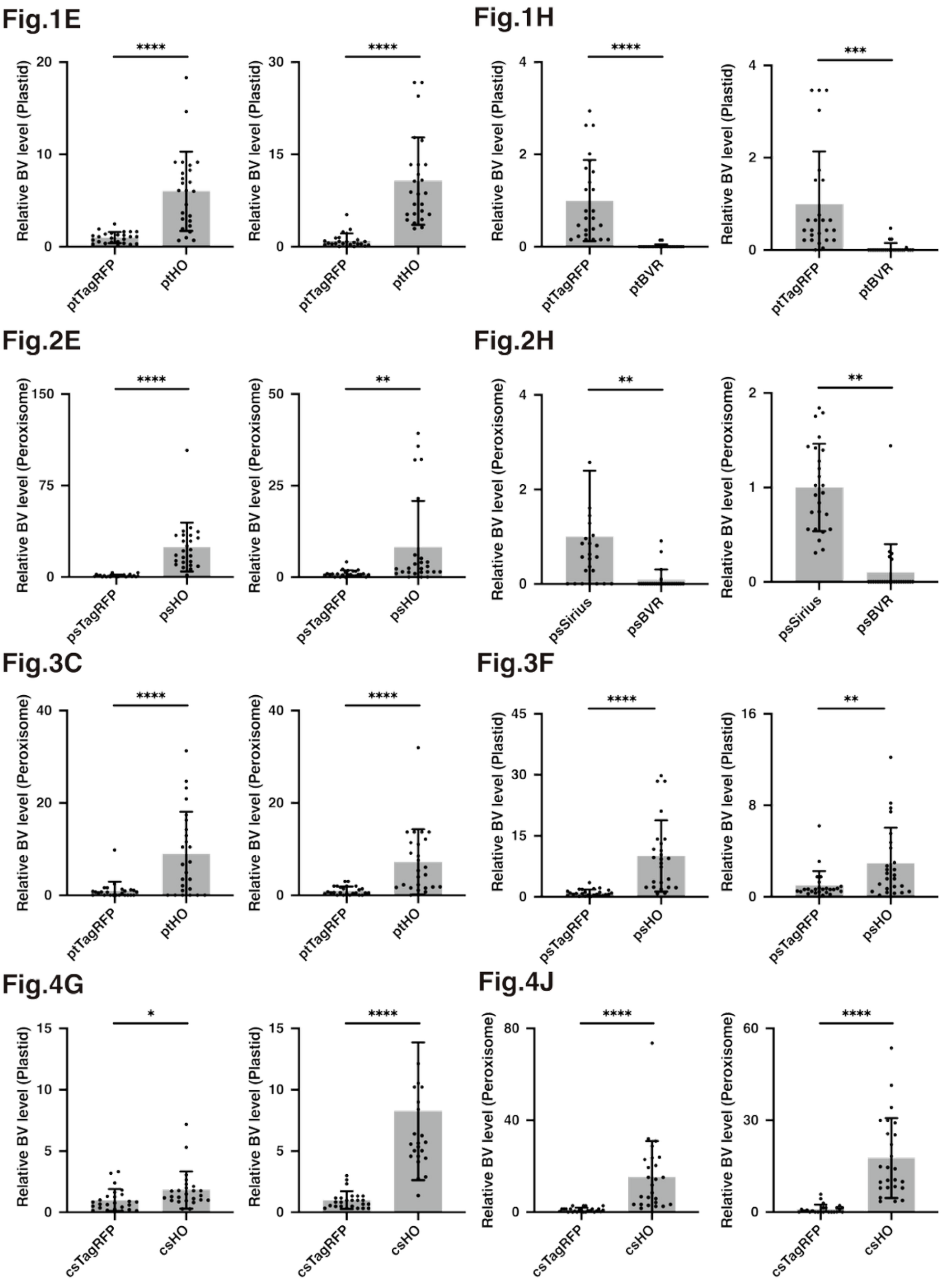

Fig. S10. Reproducibility of quantification in Figs. 1–4. Reproducibility data corresponding to Figs. 1E, 1H, 2E, 2H, 3C, 3F, 4G, and 4J are shown (two independent datasets for each panel).

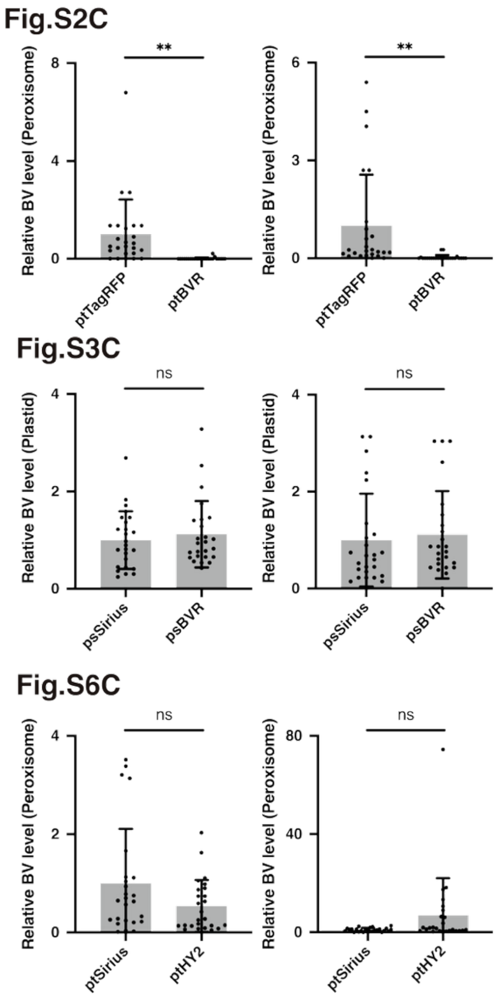

Fig. S11. Reproducibility of quantification in Supplementary Figs. S2, S3, and S6. Reproducibility data corresponding to Supplementary Figs. S2C, S3C, and S6C are shown (two independent datasets for each panel).

Biology Open • Supplementary information

Table S1. Prediction os subcellular- localization and topologies of the 132 Arabidopsis ABC transporters

Subfamily [*1]	Name [*1]	Locus	Subcellular Localization Prediction (WoLF PSORT) [*2]	Subcellular Localization Prediction (DeepLoc) [*3]		Predicted topologies (DeepTMHMM) [*4]	Uniprot ID [*5]	Uniprot Annotation [*5]	Length	Sequence
				Subcellular Localization Prediction	Predicted topologies					
A	ABCA1	At2g41700	plas: 11, chlo: 1, mito: 1, pero: 1	Cell membrane, Lysosome/Vacuole	Transmembrane	+	Q84M24	ABCA1 ABC1 AOH1 At2g41700 T32G6.22	1882	[illegible]
	ABCA2	At3g47730	plas: 11, chlo: 2, mito: 1	Cell membrane, Lysosome/Vacuole	Transmembrane	+	Q84K47	ABCA2 ATH1 At3g47730 T23J7.60	983	[illegible]
	ABCA3	At3g47740	plas: 12, mito: 1, E.R.: 1	Cell membrane, Lysosome/Vacuole	Transmembrane	+	Q1PEH6	ABCA3 ATH2 At3g47740 T23J7.70	932	[illegible]
	ABCA4	At3g47750	plas: 11, E.R.: 2, mito: 1	Cell membrane, Lysosome/Vacuole	Transmembrane	+	Q9STT8	ABCA4 ATH3 At3g47750 T23J7.80	937	[illegible]
	ABCA5	At3g47760	plas: 10, E.R.: 3, golg: 1	Cell membrane, Lysosome/Vacuole	Transmembrane	+	Q9STT7	ABCA5 ATH4 At3g47760 T23J7.90	936	[illegible]
	ABCA6	At3g47770	plas: 12, mito: 1, E.R.: 1	Lysosome/Vacuole	Transmembrane	+	Q9STT6	ABCA6 ATH5 At3g47770 T23J7.100	926	[illegible]
	ABCA7	At3g47780	plas: 10, E.R.: 2, chlo: 1, mito: 1	Cell membrane, Lysosome/Vacuole	Transmembrane	+	Q9STT5	ABCA7 ATH6 At3g47780 T23J7.110	935	[illegible]
	ABCA8	At3g47790	plas: 10, nucl: 1, vacu: 1, E.R.: 1, golg: 1	Cell membrane, Lysosome/Vacuole	Transmembrane	+	Q8LPK0	ABCA8 ATH7 At3g47790 T23J7.120	901	[illegible]
	ABCA9	At5g61730	plas: 11, E.R.: 2, golg: 1	Cell membrane, Lysosome/Vacuole	Transmembrane	+	Q9FLT5	ABCA9 ATH11 At5g61730 MAC9.4	950	[illegible]
	ABCA10	At5g61740	plas: 10, E.R.: 2, mito: 1, pero: 1	Lysosome/Vacuole	Transmembrane	+	Q9FLT4	ABCA10 ATH14 At5g61740 MAC9.5	909	[illegible]
	ABCA11	At5g61690	plas: 11, chlo: 1, mito: 1, E.R.: 1	Cell membrane, Lysosome/Vacuole	Transmembrane	+	Q9FKF2	ABCA11 ATH15 At5g61690 K11J9.22	953	[illegible]
	ABCA12	At5g61700	plas: 9, E.R.: 3, mito: 1, golg: 1	Lysosome/Vacuole	Transmembrane	+	Q9FLT8	ABCA12 ATH16 At5g61700 K11J9.23 MAC9.1	917	[illegible]
B	ABCB1	At2g36910	plas: 13, vacu: 1	Cell membrane, Lysosome/Vacuole	Transmembrane	+	Q9ZR72	ABCB1 MDR1 PGP1 At2g36910 T1J8.9	1286	[illegible]
	ABCB2	At4g25960	plas: 13, vacu: 1	Cell membrane	Transmembrane	+	Q8LPK2	ABCB2 MDR2 PGP2 At4g25960 F20B18.70	1273	[illegible]
	ABCB3	At4g01820	plas: 7, vacu: 4, golg: 2, cyto: 1	Cell membrane	Transmembrane	+	Q9SYI2	ABCB3 MDR3 PGP3 At4g01820 T7B11.8	1229	[illegible]
	ABCB4	At2g47000	plas: 10, golg: 2, vacu: 1, E.R.: 1	Cell membrane	Transmembrane	+	O80725	ABCB4 MDR4 PGP4 At2g47000 F14M4.17	1286	[illegible]
	ABCB5	At4g01830	plas: 6, vacu: 5, golg: 2, E.R.: 1	Cell membrane	Transmembrane	+	Q9SYI3	ABCB5 MDR5 PGP5 At4g01830 T7B11.9	1230	[illegible]
	ABCB6	At2g39480	plas: 13, nucl: 1	Cell membrane, Lysosome/Vacuole	Transmembrane	+	Q8LPT1	ABCB6 MDR6 PGP6 At2g39480 F12L6.14	1407	[illegible]
	ABCB7	At5g46540	plas: 12, mito: 1, E.R.: 1	Cell membrane	Transmembrane	+	Q9FHF1	ABCB7 MDR7 PGP7 At5g46540 K11I1.13	1248	[illegible]
	ABCB8	At3g30875	plas: 12, mito: 1, pero: 1	Cell membrane, Lysosome/Vacuole	Transmembrane	+	Q9LHK4	ABCB8 MDR22 PGP8 At3g30875 MJI6.16	1241	[illegible]
	ABCB9	At4g18050	plas: 13, vacu: 1	Cell membrane	Transmembrane	+	Q9M0M2	ABCB9 MDR9 PGP9 At4g18050 F15J5.20	1236	[illegible]
	ABCB10	At1g10680	plas: 14	Cell membrane, Lysosome/Vacuole	Transmembrane	+	Q9SGY1	ABCB10 MDR10 PGP10 At1g10680 F20B24.12	1227	[illegible]
	ABCB11	At1g02520	plas: 10, golg: 2, vacu: 1, E.R.: 1	Cell membrane	Transmembrane	+	Q9FWX7	ABCB11 MDR8 PGP11 At1g02520 T14P4.15	1278	[illegible]
	ABCB12	At1g02530	plas: 10, golg: 2, vacu: 1, E.R.: 1	Cell membrane	Transmembrane	+	Q9FWX8	ABCB12 MDR16 PGP12 At1g02530 T14P4.14	1273	[illegible]
	ABCB13	At1g27940	plas: 13, vacu: 1	Cell membrane, Lysosome/Vacuole	Transmembrane	+	Q9C7F8	ABCB13 MDR15 PGP13 At1g27940 F13K9.5	1245	[illegible]
	ABCB14	At1g28010	plas: 8, vacu: 3, golg: 3	Cell membrane, Lysosome/Vacuole	Transmembrane	+	Q9C7F2	ABCB14 MDR12 PGP14 At1g28010 F13K9.11	1247	[illegible]
	ABCB15	At3g28345	plas: 12, vacu: 1, E.R.: 1		Transmembrane	+	Q9LHD1	ABCB15 MDR13 PGP15 At3g28345 MZF16.16	1240	[illegible]
	ABCB16	At3g28360	plas: 12, vacu: 1, E.R.: 1	Cell membrane, Lysosome/Vacuole	Transmembrane	+	Q9LSJ8	ABCB16 MDR18 PGP16 At3g28360 MFJ20.4	1228	[illegible]
	ABCB17	At3g28380	plas: 13, vacu: 1	Cell membrane	Transmembrane	+	Q9LSJ6	ABCB17 MDR19 PGP17 At3g28380 MFJ20.6	1240	[illegible]
	ABCB18	At3g28390	plas: 12, E.R.: 2	Cell membrane, Lysosome/Vacuole	Transmembrane	+	Q9LSJ5	ABCB18 MDR20 PGP18 At3g28390 MFJ20.7	1225	[illegible]
	ABCB19	At3g28860	plas: 13, E.R.: 1	Cell membrane	Transmembrane	+	Q9LJX0	ABCB19 MDR1 MDR11 PGP19 At3g28860 MLD15.2	1252	[illegible]
	ABCB20	At3g55320	plas: 13, nucl: 1	Cell membrane, Lysosome/Vacuole	Transmembrane	+	Q9M3B9	ABCB20 MDR14 PGP20 At3g55320 T26I12.200	1408	[illegible]
	ABCB21	At3g62150	plas: 10, golg: 2, vacu: 1, E.R.: 1	Cell membrane	Transmembrane	+	Q9M1Q9	ABCB21 MDR17 PGP21 At3g62150 T17J13.110	1296	[illegible]
	ABCB22	At3g28415	plas: 11, vacu: 2, chlo: 1	Cell membrane	Transmembrane	+	Q9LSJ2	ABCB22 MDR21 PGP22 At3g28415 MFJ20.11	1229	[illegible]
	ABCB23	At4g28630	chlo: 11, mito: 2, plas: 1	Mitochondrion	Transmembrane	+	Q9FUT3	ABCB23 ATM1 STA2 At4g28630 T5F17.80	678	[illegible]
	ABCB24	At4g28620	chlo: 10, mito: 3, plas: 1	Mitochondrion	Transmembrane	+	Q9M0G9	ABCB24 ATM2 STA3 At4g28620 T5F17.70	680	[illegible]
	ABCB25	At5g58270	chlo: 8, plas: 4, cyto: 1, mito: 1	Mitochondrion	Transmembrane	+	Q9LVM1	ABCB25 ATM3 STA1 At5g58270 MCK7.14	728	[illegible]
	ABCB26	At1g70610	plas: 6, chlo: 4, E.R.: 2, mito: 1, pero: 1	Plastid	Transmembrane	+	Q8RY46	ABCB26 TAP1 At1g70610 F24J13.18 F5A18.21	700	[illegible]
	ABCB27	At5g39040	E.R.: 6, plas: 5.5, cyto_plas: 3.5, vacu: 2	Lysosome/Vacuole	Transmembrane	+	Q0WML0	ABCB27 ALS1 TAP2 At5g39040 MXF12.50	644	[illegible]
	ABCB28	At4g25450	chlo: 11, mito: 2, cyto: 1	Plastid	Transmembrane	+	Q8LPQ6	ABCB28 NAP8 At4g25450 M7J2.180	714	[illegible]
	ABCB29	At5g03910	plas: 7, chlo: 6, pero: 1	Plastid	Transmembrane	+	Q9LZB8	ABCB29 ATH12 At5g03910 F8F6.120	634	[illegible]
C	ABCC1	At1g30400	plas: 12, vacu: 1, E.R.: 1	Cell membrane, Lysosome/Vacuole	Transmembrane	+	Q9C8G9	ABCC1 EST1 MRP1 At1g30400 T4K22.12	1622	[illegible]
	ABCC2	At2g34660	plas: 13, vacu: 1	Cell membrane, Lysosome/Vacuole	Transmembrane	+	Q42093	ABCC2 EST4 MRP2 At2g34660 T29F13.13	1623	[illegible]
	ABCC3	At3g13080	plas: 11, E.R.: 2, vacu: 1	Cell membrane, Lysosome/Vacuole	Transmembrane	+	Q9LK64	ABCC3 EST2 MRP3 At3g13080 MJG19.3	1514	[illegible]
	ABCC4	At2g47800	plas: 13, vacu: 1	Cell membrane, Lysosome/Vacuole	Transmembrane	+	Q7DM58	ABCC4 EST3 MRP4 At2g47800 F17A22.19	1516	[illegible]
	ABCC5	At1g04120	plas: 9, vacu: 3, E.R.: 2	Cell membrane, Lysosome/Vacuole	Transmembrane	+	Q7GB25	ABCC5 MRP5 At1g04120 F20D22.11	1514	[illegible]
	ABCC6	At3g13090	plas: 9, vacu: 3, mito: 1, E.R.: 1	Cell membrane, Lysosome/Vacuole	Transmembrane	+	Q8VZZ4	ABCC6 MRP6 MRP8 At3g13090 MJG19.4	1466	[illegible]
	ABCC7	At3g13100	plas: 12, vacu: 2	Cell membrane, Lysosome/Vacuole	Transmembrane	+	Q9LK62	ABCC7 MRP7 At3g13100 MJG19.5	1493	[illegible]
	ABCC8	At3g21250	plas: 9, vacu: 3, E.R.: 2	Cell membrane, Lysosome/Vacuole	Transmembrane	+	Q8LGU1	ABCC8 MRP6 MRP8 At3g21250 MXL8.11	1464	[illegible]
	ABCC9	At3g60160	plas: 12, vacu: 2	Cell membrane, Lysosome/Vacuole	Transmembrane	+	Q9M1C7	ABCC9 MRP9 At3g60160 T2O9.140	1506	[illegible]
	ABCC10	At3g59140	plas: 13, chlo: 1	Cell membrane, Lysosome/Vacuole	Transmembrane	+	Q9LYS2	ABCC10 MRP10 MRP14 At3g59140 F17J16.190	1453	[illegible]
	ABCC11	At1g30420	plas: 11, nucl: 1, vacu: 1, E.R.: 1	Cell membrane, Lysosome/Vacuole	Transmembrane	+	Q9C8H1	ABCC11 MRP11 MRP12 At1g30420 F26G16.1 T4K22.1	1495	[illegible]
	ABCC12	At1g30410	plas: 12, vacu: 1, E.R.: 1	Cell membrane, Lysosome/Vacuole	Transmembrane	+	Q9C8H0	ABCC12 MRP12 MRP13 At1g30410 T4K22.13	1495	[illegible]
	ABCC13	At2g07680	plas: 9, vacu: 2, E.R.: 2, cyto: 1	Lysosome/Vacuole	Transmembrane	+	Q9SKX0	ABCC13 MRP11 MRP13 At2g07680 T5E7.1	1410	[illegible]
	ABCC14	At3g62700	plas: 14	Cell membrane, Lysosome/Vacuole	Transmembrane	+	Q9LZJ5	ABCC14 MRP10 MRP14 At3g62700 F26K9.130	1539	[illegible]
	ABCC15	At3g60970	plas: 9, vacu: 3, extr: 1, E.R.: 1	Cell membrane, Lysosome/Vacuole	Transmembrane	+	Q7FB56	ABCC15 MRP15 At3g60970 T27I15.60	1053	[illegible]
D	ABCD1	At4g39850	plas: 8, chlo: 2, mito: 2, E.R.: 1, pero: 1	Mitochondrion, Peroxisome	Transmembrane	+	Q94FB9	ABCD1 ACN2 CTS PED3 PMP2 PXA1 At4g39850 T5J17.20	1337	[illegible]
	ABCD2	At1g54350	chlo: 6, plas: 5, vacu: 1, E.R.: 1, pero: 1	Plastid	Transmembrane	+	Q6NLC1	ABCC2 PMP1 At1g54350 F20D21.17	706	[illegible]
E	ABCE1	At3g13640	cyto: 8, nucl: 4, chlo: 1, mito: 1	Cytoplasm	Soluble	-	Q9LID6	ABCE1 RLI1 At3g13640 MMM17.7	603	[illegible]
	ABCE2	At4g19210	cyto: 9, nucl: 4, cyto_mito: 1	Cytoplasm	Soluble	-	Q8LPJ4	ABCE2 RLI2 At4g19210 T18B16.180	605	[illegible]
	ABCE3	At4g30300	nucl: 6.5, cyto_nucl: 6, cyto: 4.5, extr: 2, mito: 1	Cytoplasm	Soluble	-	Q9M0D0	ABCE3 NAP15 RLI3 At4g30300 F17I23.360	181	[illegible]
F	ABCF1	At5g60790	nucl: 5, chlo: 3, mito: 3, golg: 2, cyto: 1	Cytoplasm	Soluble	-	Q9FJH6	ABCF1 GCN1 At5g60790 MAE1.10	595	[illegible]
	ABCF2	At5g09930	chlo: 12.5, chlo_mito: 7.5, mito: 1.5	Plastid	Soluble	-	Q9FIB4	ABCF2 GCN2 At5g09930 MYH9.14	678	[illegible]
	ABCF3	At1g64550	cyto: 13, chlo: 1	Cytoplasm	Soluble	-	Q8H0V6	ABCF3 GCN3 At1g64550 F1N19.11	715	[illegible]
	ABCF4	At3g54540	cyto: 7, nucl: 4, chlo: 1, plas: 1, cysk: 1	Cytoplasm	Soluble	-	Q9M1H3	ABCF4 GCN4 At3g54540 T14E10.110	723	[illegible]
	ABCF5	At5g64840	chlo: 14	Plastid	Soluble	-	Q9LV93	ABCF5 GCN5 At5g64840 MXK3.6	692	[illegible]
G	ABCG1	At2g39350	plas: 10, E.R.: 2, vacu: 1, golg: 1	Cell membrane, Lysosome/Vacuole	Transmembrane	+	O80946	ABCG1 WBC1 At2g39350 T16B24.1	740	[illegible]
	ABCG2	At2g37360	plas: 10, E.R.: 2, nucl: 1, mito: 1	Cell membrane, Lysosome/Vacuole	Transmembrane	+	Q9ZUT0	ABCG2 WBC2 At2g37360 F3G5.15	755	[illegible]
	ABCG3	At2g28070	plas: 7, vacu: 3, E.R.: 3, golg: 1	Cell membrane	Transmembrane	+	Q9ZUU9	ABCG3 WBC3 At2g28070 F24D13.14	730	[illegible]
	ABCG4	At4g25750	plas: 10, E.R.: 2, chlo: 1, cyto: 1	Cell membrane, Endoplasmic reticulum, Lysosome/Vacuole	Transmembrane	+	Q9SW08	ABCG4 WBC4 At4g25750 F14M19.30	577	[illegible]
	ABCG5	At2g13610	plas: 7, E.R.: 4, vacu: 2, mito: 1	Cell membrane, Lysosome/Vacuole	Transmembrane	+	Q9SIT6	ABCG5 WBC5 At2g13610 T10F5.15	649	[illegible]
	ABCG6	At5g13580	plas: 8, vacu: 3, chlo: 1, mito: 1, E.R.: 1	Cell membrane, Lysosome/Vacuole	Transmembrane	+	Q9FNB5	ABCG6 WBC6 At5g13580 MSH12.4	727	[illegible]
	ABCG7	At2g01320	plas: 9, vacu: 2, E.R.: 2, chlo: 1	Plastid, Endoplasmic reticulum, Peroxisome	Transmembrane	+	Q9ZU35	ABCG7 WBC7 At2g01320 F10A8.20	725	[illegible]
	ABCG8	At5g52860	plas: 5, E.R.: 3, cyto: 2, vacu: 2, mito: 1, golg: 1	Cell membrane, Endoplasmic reticulum, Lysosome/Vacuole	Transmembrane	+	Q9FLX5	ABCG8 WBC8 At5g52860 MXC20.8	589	[illegible]
	ABCG9	At4g27420	plas: 9, vacu: 2, E.R.: 2, golg: 1	Cell membrane	Transmembrane	+	Q9SZR9	ABCG9 WBC9 At4g27420 F27G19.20	638	[illegible]
	ABCG10	At1g53270	plas: 8, vacu: 3, golg: 2, E.R.: 1	Cell membrane, Endoplasmic reticulum	Transmembrane	+	Q9MAH4	ABCG10 WBC10 At1g53270 F12M16.17	590	[illegible]
	ABCG11	At1g17840	plas: 6, vacu: 3, E.R.: 2, cyto: 1, mito: 1, golg: 1	Cell membrane, Lysosome/Vacuole	Transmembrane	+	Q8RXN0	ABCG11 COF1 DSO PEL1 WBC11 At1g17840 F2H15.7	703	[illegible]
	ABCG12	At1g51500	plas: 9, nucl: 2, mito: 1, vacu: 1, E.R.: 1	Cell membrane, Lysosome/Vacuole	Transmembrane	+	Q9C8K2	ABCG12 CER5 D3 WBC12 At1g51500 F5D21.6	687	[illegible]
	ABCG13	At1g51460	plas: 8, E.R.: 3, vacu: 2, nucl: 1	Cell membrane, Endoplasmic reticulum	Transmembrane	+	Q9C8J8	ABCG13 WBC13 At1g51460 F5D21.8	678	[illegible]
	ABCG14	At1g31770	chlo: 6, nucl: 3.5, cysk_nucl: 2.5, mito: 1, plas: 1, vacu: 1, E.R.: 1	Cell membrane	Transmembrane	+	Q9C6W5	ABCG14 INT211 WBC14 At1g31770 F27M3.2 F5M6.22	648	[illegible]
	ABCG15	At3g21090	plas: 11, nucl: 1, mito: 1, golg: 1	Cell membrane, Lysosome/Vacuole	Transmembrane	+	Q8RWI9	ABCG15 WBC15 WBC22 At3g21090 MSA6.13	691	[illegible]
	ABCG16	At3g55090	plas: 13, vacu: 1	Cell membrane, Lysosome/Vacuole	Transmembrane	+	Q9M2V7	ABCG16 WBC16 At3g55090 T15C9.80	736	[illegible]
	ABCG17	At3g55100	plas: 7, vacu: 4, E.R.: 2, golg: 1	Cell membrane, Lysosome/Vacuole	Transmembrane	+	Q9M2V6	ABCG17 WBC17 At3g55100 T15C9.100	662	[illegible]
	ABCG18	At3g55110	plas: 9, vacu: 2, E.R.: 2, golg: 1	Cell membrane, Lysosome/Vacuole	Transmembrane	+	Q9M2V5	ABCG18 WBC18 At3g55110 T15C9.110	708	[illegible]
	ABCG19	At3g55130	plas: 8, E.R.: 4, chlo: 1, mito: 1	Cell membrane, Lysosome/Vacuole	Transmembrane	+	Q9M3D6	ABCG19 WBC19 At3g55130 T26I12.10	725	[illegible]
	ABCG20	At3g53510	plas: 12, nucl: 1, mito: 1	Cell membrane, Lysosome/Vacuole	Transmembrane	+	Q9LFG8	ABCG20 WBC20 At3g53510 F4P12.210	739	[illegible]
	ABCG21	At3g25620	plas: 8, golg: 2, nucl: 1, mito: 1, vacu: 1, E.R.: 1	Cell membrane	Transmembrane	+	Q7XA72	ABCG21 WBC21 At3g25620 T5M7.6	672	[illegible]
	ABCG22	At5g06530	plas: 6, E.R.: 3, chlo: 2, mito: 2, pero: 1	Cell membrane	Transmembrane	+	Q93YS4	ABCG22 WBC23 At5g06530 F15M7.6	751	[illegible]
	ABCG23	At5g19410	plas: 9, vacu: 2, E.R.: 2, cyto: 1	Cell membrane, Endoplasmic reticulum, Lysosome/Vacuole	Transmembrane	+	Q3E9B8	ABCG23 WBC24 At5g19410 F7K24.160	624	[illegible]
	ABCG24	At1g53390	plas: 9.5, golg_plas: 6.5, golg: 2.5, chlo: 1, E.R.: 1	Cell membrane	Transmembrane	+	Q9MAG3	ABCG24 WBC25 At1g53390 F12M16.28	1109	[illegible]
	ABCG25	At1g71960	plas: 8, vacu: 3, E.R.: 2, golg: 1	Cell membrane	Transmembrane	+	Q84TH5	ABCG25 WBC26 At1g71960 F17M19.11	662	[illegible]
	ABCG26	At3g13220	plas: 7, E.R.: 3, vacu: 2, chlo: 1, pero: 1	Cell membrane	Transmembrane	+	Q9LK50	ABCG26 WBC27 At3g13220 MJG19.19	685	[illegible]
	ABCG27	At3g52310	plas: 10, vacu: 2, nucl: 1, E.R.: 1	Cell membrane	Transmembrane	+	Q9FT51	ABCG27 WBC28 At3g52310 T25B15.80	737	[illegible]
	ABCG28	At5g60740	plas: 9.5, golg_plas: 7, golg: 3.5, E.R.: 1	Cell membrane, Lysosome/Vacuole	Transmembrane	+	Q9FF46	ABCG28 WBC29 At5g60740 MUP24.16	1109	[illegible]
	ABCG29	At3g16340	plas: 11, vacu: 2, E.R.: 1	Cell membrane	Transmembrane	+	Q94A18	ABCG29 PDR1 At3g16340 MYA6.14 T02O04.17	1416	[illegible]
	ABCG30	At4g15230/At4g15220	plas: 9, vacu: 3, golg: 2	Cell membrane	Transmembrane	+	Q8GZ52	ABCG30 PDR2 At4g15230/At4g15220 dl3660w/dl3655w FCAALL.241	1400	[illegible]
	ABCG31	At2g29940	plas: 11, E.R.: 2, nucl: 1	Cell membrane	Transmembrane	+	Q7PC88	ABCG31 PDR3 At2g29940 F23F1.14	1426	[illegible]
	ABCG32	At2g26910	plas: 10, vacu: 2, E.R.: 2	Cell membrane	Transmembrane	+	O81016	ABCG32 PDR4 PEC1 At2g26910 F12C20.5	1420	[illegible]
	ABCG33	At2g37280	plas: 13, E.R.: 1	Cell membrane	Transmembrane	+	Q9ZUT8	ABCG33 PDR5 At2g37280 F3G5.7	1413	[illegible]
	ABCG34	At2g36380	plas: 12, nucl: 1, E.R.: 1	Cell membrane	Transmembrane	+	Q7PC87	ABCG34 PDR6 At2g36380 F1O11.1	1453	[illegible]
	ABCG35	At1g15210	plas: 9, golg: 3, cyto: 1, E.R.: 1	Cell membrane	Transmembrane	+	Q7PC86	ABCG35 PDR7 At1g15210 F9L1.15	1442	[illegible]
	ABCG36	At1g59870	plas: 11, chlo: 1, mito: 1, E.R.: 1	Cell membrane	Transmembrane	+	Q9XIE2	ABCG36 PDR8 PEN3 At1g59870 F23H11.19	1469	[illegible]
	ABCG37	At3g53480	plas: 10, vacu: 3, E.R.: 1	Cell membrane	Transmembrane	+	Q9LFH0	ABCG37 ETA4 PDR12 PDR9 PIS1 At3g53480 F4P12.180	1450	[illegible]
	ABCG38	At3g30842	plas: 12, E.R.: 2	Cell membrane	Transmembrane	+	Q7PC85	ABCG38 PDR10 At3g30842 MJI6.3	1418	[illegible]
	ABCG39	At1g66950	plas: 10, vacu: 2, E.R.: 2	Cell membrane	Transmembrane	+	Q7PC84	ABCG39 PDR11 PDR13 At1g66950 F1O19.3 T4O24.9	1454	[illegible]
	ABCG40	At1g15520	plas: 12.5, cyto_plas: 7, E.R.: 1	Cell membrane	Transmembrane	+	Q9M9E1	ABCG40 PDR12 PDR9 At1g15520 T16N11.3	1423	[illegible]
	ABCG41	At4g15215	plas: 11, E.R.: 2, vacu: 1	Cell membrane	Transmembrane	+	Q7PC83	ABCG41 PDR13 At4g15215 FCAALL.459	1397	[illegible]
	ABCG42	At4g15233	plas: 10, E.R.: 2, nucl: 1, vacu: 1	Cell membrane	Transmembrane	+	Q7PC82	ABCG42 PDR14 At4g15233 FCAALL.460	1392	[illegible]
	ABCG43	At4g15236	plas: 9, vacu: 2, nucl: 1, E.R.: 1, golg: 1	Cell membrane	Transmembrane	+	Q7PC81	ABCG43 PDR15 At4g15236 FCAALL.461	1390	[illegible]
I	ABCI1	At1g63270	chlo: 9, mito: 5	Cytoplasm, Nucleus	Soluble	-	Q9C8T1	ABCI1 CCMA NAP10 At1g63270 F9N12.11	229	[illegible]
	ABCI2	AtMg00110	plas: 7, vacu: 3, golg: 3, chlo: 1	Endoplasmic reticulum	Transmembrane	+	P93280	CCMB ABCI2 CCB2 CCB206 AtMg00110	206	[illegible]
	ABCI3	AtMg00900	plas: 9, golg: 2, chlo: 1, vacu: 1, E.R.: 1	Cell membrane, Endoplasmic reticulum	Transmembrane	+	P92527	CCMC ABCI3 ABCI4 ABCI5 CCB256 CCB3 AtMg00900	256	[illegible]
	ABCI4	At2g07681.1	plas: 8, nucl: 3, E.R.: 2, golg: 1	Endoplasmic reticulum	Transmembrane	+	F4IMA2	ABCI4 ATP-binding cassette I4 At2g07681	736	[illegible]
	ABCI5	At2g07771.2	plas: 8, vacu: 3, golg: 2, chlo: 1	Cell membrane, Endoplasmic reticulum	Transmembrane	+	F4IL76	ABCI5 ATP-binding cassette I5 ccb256 CCMC ccmC At2g07771	256	[illegible]
	ABCI6	At3g10670	chlo: 14	Plastid	Soluble	-	Q9CAF5	ABCI6 NAP7 At3g10670 F13M14.4 F18K10.29 T7M13.25	338	[illegible]
	ABCI7	At1g32500	chlo_mito: 6, chlo: 5.5, mito: 5.5, nucl: 2, cyto: 1	Plastid	Soluble	-	Q9LQK7	ABCI7 NAP6 At1g32500 F5D14.28	475	[illegible]
	ABCI8	At4g04770	chlo: 11.5, chlo_mito: 6.5, nucl: 1, cysk: 1	Plastid	Soluble	-	Q9ZS97	ABCI8 LAF6 NAP1 At4g04770 T4B21.16	557	[illegible]
	ABCI9	At5g44316	chlo: 8.5, chlo_mito: 6, mito: 2.5, nucl: 2, extr: 1	Plastid	Soluble	-	Q3E8H7	ABCI9 At5g44316 K9L2.9	470	[illegible]
	ABCI10	At4g33460	chlo: 12, mito: 2	Plastid	Soluble	-	Q8H1R4	ABCI10 EMB2751 NAP13 At4g33460 F17M5.220	271	[illegible]
	ABCI11	At5g14100	chlo: 8, cyto: 3, cysk: 2, nucl: 1	Plastid	Peripheral, Soluble	-	Q8LEF6	ABCI11 NAP14 At5g14100 MUA22.10	278	[illegible]
	ABCI12	At3g21580	plas: 8, chlo: 4, E.R.: 2	Plastid	Transmembrane	+	Q944H2	ABCI12 At3g21580 MIL23.15	391	[illegible]
	ABCI13	At1g65410	chlo: 10.5, chlo_mito: 7.33333, mito: 3, cyto_mito: 2.33333	Plastid	eripheral, Transmembra	-	Q9AT00	TGD3 ABCI13 NAP11 At1g65410 T8F5.19	345	[illegible]
	ABCI14	At1g19800	plas: 8, E.R.: 4, mito: 1, pero: 1	Plastid	Transmembrane	+	Q8L4R0	TGD1 ABCI14 At1g19800 F14P1.27	350	[illegible]
	ABCI15	At3g20320	chlo: 13, extr: 1	Plastid	eripheral, Transmembra	+	Q9LTR2	TGD2 ABCI15 At3g20320 MQC12.6	381	[illegible]
	ABCI16	At2g37330	vacu: 13, golg: 1	Lysosome/Vacuole	Transmembrane	+	Q9ZUT3	ALS3 ABCI16 At2g37330 F3G5.12	273	[illegible]
	ABCI17	At1g67940	golg: 5, cyto: 3, chlo: 2, vacu: 2, plas: 1, E.R.: 1	Cytoplasm	Peripheral	-	Q9C9W0	ABCI17 NAP3 At1g67940 T23K23.21	263	[illegible]
	ABCI18	At1g03900	nucl: 9, cyto: 4, golg: 1	Cytoplasm	Peripheral, Soluble	-	Q681Q7	Y1390_ARATH	272	[illegible]
	ABCI19	At1g03905	cyto: 4.5, cysk: 4, cyto_nucl: 4, nucl: 2.5, chlo: 2, plas: 1	Cytoplasm, Nucleus	Soluble	-	Q3EDJ0	ABCI19 NAP4 At1g03905 F21M11.17	290	[illegible]
	ABCI20	At5g02270	cyto: 10, cysk: 2, chlo: 1, E.R.: 1	Cytoplasm	Soluble	-	Q9LZ98	ABCI20 NAP9 At5g02270 T1E22.30	328	[illegible]
	ABCI21	At5g44110	cyto: 4, nucl: 3, cysk: 3, plas: 2, chlo: 1, E.R._vacu: 1	Cytoplasm, Nucleus	Soluble	-	Q9XF19	ABCI21 NAP2 POP1 At5g44110 MLN1.3	282	[illegible]
Other	-	At1g71330	plas: 4, vacu: 4, extr: 2, E.R.: 2, nucl: 1, golg: 1	Cell membrane, Endoplasmic reticulum, Lysosome/Vacuole, Golgi apparatus	Transmembrane	+	Q9FVV9	NAP5 At1g71330 F3I17.2	324	[illegible]
	-	At2g37010	plas: 9.5, golg_plas: 6.5, golg: 2.5, vacu: 1, E.R.: 1	Cell membrane, Lysosome/Vacuole	Transmembrane	+	Q9SJK6	WBC30 NAP12 At2g37010 T1J8.19	1082	[illegible]

[*1] Verrier et al., 2008

[*2] WoLF PSORT [https://wolfpsort.hgc.jp/]: plas (plasmamembrane), cyto (cytosol), chlo (chloroplast/plastid), pero (peroxisome), mito (mitochondrion), nucl (nucleus), E.R. (endoplasmic reticulum), golg (Golgi apparatus), vacu (vacuole), cysk (cytoskelton), extr (extracellular)

[*3] DeepLoc 2.1 [https://services.healthtech.dtu.dk/services/DeepLoc-2.1/]

[*4] Deep TMHMM 1.0 [https://services.healthtech.dtu.dk/services/DeepTMHMM-1.0/]

[*5] UniProt database [https://www.uniprot.org/]

Biology Open • Supplementary information

Table S2. Primers used in this study.

Number	Designation	Sequence (5' to 3')
#1	RBCS-attB1	GGGGACAAGTTTGTACAAAAAAGCAGGCTTCATGGCTTCCTCTATGCTC
#2	sfGFP-smURFP-attB2	GGGGACCACTTTGTACAAGAAAGCTGGGTCTTAGCTCATAGCCTTAATAATG
#3	sfGFP-smURFP-attB1	GGGGACAAGTTTGTACAAAAAAGCAGGCTTCATGGTGAGCAAGGGCGAGGAG
#4	smURFP-SKL-attB2	GGGGACCACTTTGTACAAGAAAGCTGGGTCTCACAACTTGGAGCTCATAGCCTTAATAAT
#5	AtCYSC1-attB1	GGGGACAAGTTTGTACAAAAAAGCAGGCTTCATGGCCTCTGTTTCA
#6	AtCYSC1(1–55)-linker-R	ACCGCCGCTACCGCCCAGAGAGGCGTCACGTTTGGC
#7	linker-sfGFP-F	GGCGGTAGCGGCGGTATGGTGAGCAAGGGCGAG
#8	SP(1–25)-attB1	GGGGACAAGTTTGTACAAAAAAGCAGGCTTCATGGCCAGACTCACAAGCATCATTGCCCTC
#9	SP(1–25)-sfGFP-R	GCCCTTGCTCACCATTGTGCGGTAGGCGTA
#10	SP(1–25)-sfGFP-F	TACGCCTACCGCACAATGGTGAGCAAGGGC
#11	smURFP-HDEL-attB2	GGGGACCACTTTGTACAAGAAAGCTGGGTCTTAGAGCTCGTCGTGGCTCATAGCCTTAAT
#12	TagRFP-T-attB1	GGGGACAAGTTTGTACAAAAAAGCAGGCTTCATGGTGTCTAAGGGCGAA
#13	TagRFP-T-attB2	GGGGACCACTTTGTACAAGAAAGCTGGGTCTCAATTAAGTTTGTGCCCC
#14	TagRFP-SKL-attB2	GGGGACCACTTTGTACAAGAAAGCTGGGTTTAGAGCTCGTCGTGATTAAGTTTGTGCCC
#15	Sirius-SKL-attB2	GGGGACCACTTTGTACAAGAAAGCTGGGTCTCACAACTTGGACTTGTACAGCTCGTCC
#16	RBCS-Linker-R	GCCCTTGCTCACCATGGAATCGGTAAGGTCAGG
#17	Linker-Sirius-F	ATGGTGAGCAAGGGCGAGGAGCTGTTCACC
#18	Sirius-attB2-1	GGGGACCACTTTGTACAAGAAAGCTGGGTCTCACTTGTACAGCTCGTCC
#19	AtHO1-attB1-F	GGGGACAAGTTTGTACAAAAAAGCAGGCTTCATGGCGTATTTAGCT
#20	AtHO1-linker-R	ACCGCCGCTACCGCCGGACAATATGAGACG
#21	linker-TagRFP-T-F1	GGCGGTAGCGGCGGTATGGTGTCTAAGGGC
#22	attB1-HO1(55–)	GGGGACAAGTTTGTACAAAAAAGCAGGCTTCATGGCGGCTACTACTGCG
#23	attB1-ratBVRA-F	GGGGACAAGTTTGTACAAAAAAGCAGGCTATGGATGCCGAGCCAAAG
#24	ratBVRA-SKL-attB2	GGGGACCACTTTGTACAAGAAAGCTGGGTCCAACTTGGACTTGTCATCGTCATCCTTG
#25	HY2-attB1	GGGGACAAGTTTGTACAAAAAAGCAGGCTTCATGGCTTTATCAATG
#26	HY2-linker-R	ACCGCCGCTACCGCCGCCGATAAATTGTCC
#27	linker-Sirius-F	GGCGGTAGCGGCGGTATGGTGAGCAAGGGCGAG
#28	Sirius-attB2-2	GGGGACCACTTTGTACAAGAAAGCTGGGTCTTACTTGTACAGCTCGTCC
#29	attB1-AtFd2(53–148)	GGGGACAAGTTTGTACAAAAAAGCAGGCTTCATGGCTACATACAAGGTC
#30	AtFb2-linker-R	CCTCCTGAGCCTCCAACAATGTCTTCTTCTTT
#31	Linker-TagRFP-T-F2	GCTCAGGAGGCTCCATGGTGTCTAAGGGCGAA

Table S3. pGWT35S vectors constructed in this study.

Plasmid name	Description	Protein designation in this study
pGWT35S-RBCS(1–79)-sfGFP-smURFP	Plastid-targeted sfGFP-smURFP	$PT^{sfGFP\text{-}smURFP}$
pGWT35S-sfGFP-smURFP	Cytosol-targeted sfGFP-smURFP	$CS^{sfGFP\text{-}smURFP}$
pGWT35S-sfGFP-smURFP-SKL	Peroxisome-targeted sfGFP-smURFP	$PS^{sfGFP\text{-}smURFP}$
pGWT35S-AtCYSC1(1–55)-sfGFP-smURFP	Mitochondrion-targeted sfGFP-smURFP	$MT^{sfGFP\text{-}smURFP}$
pGWT35S-SP-sfGFP-smURFP-HDEL	ER-targeted sfGFP-smURFP	$ER^{sfGFP\text{-}smURFP}$
pGWT35S-RBCS(1–79)-TagRFP-T	Plasmid-targeted TagRFP-T	ptTagRFP
pGWT35S-TagRFP-T	Cytosol-targeted TagRFP-T	csTagRFP
pGWT35S-TagRFP-T-SKL	Peroxisome-targeted TagRFP-T	psTagRFP
pGWT35S-Sirius-SKL	Peroxisome-targeted Sirius	psSirius
pGWT35S-RBCS(1–79)-Sirius	Plastid-targeted Sirius	ptSirius
pGWT35S-HO1(1–282)-TagRFP-T	Plastid-targeted HO1-TagRFP-T	ptHO
pGWT35S-HO1(55–282)-TagRFP-T	Cytosol-targeted HO1-TagRFP-T	csHO
pGWT35S-HO1(55–282)-TagRFP-T-SKL	Peroxisome-targeted HO1-TagRFP-T	psHO
pGWT35S-RBCS(1–79)-BVRA-flag	Plastid-targeted BVRA-flag	ptBVR
pGWT35S-BVRA-flag-SKL	Peroxisome-targeted BVRA-flag-SKL	psBVR
pGWT35S-HY2-Sirius	Plastid-targeted HY2-Sirius	ptHY2
pGWT35S-Fd2-TagRFP-T	Plastid-targeted Fd2-TagRFP-T	ptFd2

Biology Open • Supplementary information
